# Cerebral ischemic events in patients with pancreatic cancer: A retrospective cohort study of 17 patients and a literature review

**DOI:** 10.1097/MD.0000000000004009

**Published:** 2016-07-01

**Authors:** Mathieu Bonnerot, Lisa Humbertjean, Gioia Mione, Jean-Christophe Lacour, Anne-Laure Derelle, Jean-Charles Sanchez, Nolwenn Riou-Comte, Sébastien Richard

**Affiliations:** a Department of Neurology, Stroke Unit; b Department of Neuroradiology, University Hospital of Nancy, Nancy, France; c Department of Human Protein Sciences, University Medical Center, Geneva, Switzerland; d Centre d’Investigation Clinique Plurithématique Pierre Drouin, University Hospital of Nancy, Vandoeuvre-lès-Nancy, France.

**Keywords:** cerebral infarction, cerebrovascular events, pancreatic adenocarcinoma, pancreatic cancer, stroke

## Abstract

Stroke is a dramatic complication of pancreatic cancer with mechanisms related to oncological disease. A better description of the characteristics of cerebrovascular events would help better understand the pathogeny and protect vulnerable patients. We thus conducted a descriptive analysis of clinical, biological, and radiological features of patients from our centers and literature.

We reviewed consecutive cases of patients who presented cerebrovascular events and pancreatic cancer in 4 stroke units in Lorrain (France) between January 1, 2009 and March 31, 2015, and all reported cases of literature. We identified 17 cases in our centers and 18 reported cases. Fifty-seven per cent of patients were male. Median age was 63 ± 14 years and ranged from 23 to 81 years. All cerebral events were ischemic. At the onset of stroke, pancreatic cancer had already been diagnosed in 59% of the patients in our centers for a mean time of 5.4 months. Five of them (29%) were being treated with gemcitabine and 2 (12%) with folfirinox. Adenocarcinoma at metastatic stage was reported in 82% of cases overall. Brain imaging revealed disseminated infarctions in 64%. High median levels of D-dimer (7600 ± 5 × 10^7^ μg/L), C-reactive protein (63 ± 43 mg/L), and elevated prothrombin time (19 ± 6 seconds) were found. Thirty-six per cent of patients explored with echocardiography were diagnosed with nonbacterial thrombotic endocarditis. Ten of our patients received anticoagulant therapy as secondary stroke prevention without any documented recurrence. Nevertheless, outcome was poor with a median survival time of 28 ± 14 days after stroke onset. Cerebral ischemic events occur at advanced stages of pancreatic cancer, most likely by a thromboembolic mechanism. Disseminated infarctions and high D-dimer, C-reactive protein levels, and a high prothrombin time are the most constant characteristics found in this context. All patients should be screened for nonbacterial thrombotic endocarditis as this etiology supports the use of anticoagulant therapy.

## Abstract 2

Supplemental Digital Content is available in the text

## 1 Introduction

Thromboembolic events—a known complication of cancer—are critical for vulnerable patients and costly for the society.^[1]^ Trousseau was the first to describe venous thrombosis during his own pancreatic cancer. His name was retained to define any thromboembolic event as the first manifestation of concealed cancer.^[2]^ Since then, most reported cases and studies have described venous thrombosis in a population of patients with various oncological diseases.^[3]^ In this context, pancreatic cancer has emerged as a leading cause with venous thrombosis observed in about 35% of affected patients in Western countries, although a lower incidence of 5% is observed in Asian populations.^[4–6]^ In contrast, reports of arterial infarctions, especially at the cerebral level, are scarce. Paraneoplastic stroke has been reported to concern 0.3% of patients and still predominantly associated with pancreatic cancer.^[7–9]^ Literature more often describes cerebral infarction as Trousseau syndrome revealing concealed pancreatic cancer. It is a result of a hypercoagulable state due to both the cancer and therapies, but the pathogeny is poorly understood, rendering it difficult to establish preventive measures.^[10,11]^ A better knowledge of cerebrovascular diseases in pancreatic cancer would help understand the pathogeny, detect patients at risk of stroke, and establish preventive measures. We decided to describe consecutive cases of patients with cerebrovascular events associated with pancreatic cancer admitted in 4 stroke units during a specified period of time. We also reviewed reported cases in literature to isolate specific clinical, radiological, and biological features of stroke patients in this particular context. We discuss thrombotic mechanisms interacting with cancer cells and promoted by some chemotherapy regimens.

## 2 Methods

This was an observational retrospective multicenter study.

### 2.1 Data collection

We reviewed cases of patients with cerebrovascular events and pancreatic cancer, who were managed in 4 stroke units in the Lorrain region (northeastern France) between January 1, 2009 and March 31, 2015. Reported cases were also identified in the literature using the database MEDLINE/Pubmed for articles published in the past 30 years, with the keywords “pancreatic cancer,” pancreatic carcinoma,” “pancreatic adenocarcinoma,” “stroke,” “cerebral infarction,” “transient ischemic attack,” and “cerebral vasculitis.” Demographic data (age, sex), information about pancreatic cancer (date of diagnosis, histologic type, stage), cerebrovascular events (type, radiological features), results of etiological investigations (electrocardiogram, echocardiography, cervical and transcranial Doppler ultrasonography, and angiography), presence of other systemic infarctions on abdominal computed tomography (CT) scan, laboratory analyses (complete blood count, coagulation tests, C-reactive protein [CRP], carbohydrate antigen 19.9 [CA19.9], liver transaminase levels), treatments (antithrombotic therapies and chemotherapy), and outcome were retrospectively collected. Brain diffusion-weighted magnetic resonance imaging and CT scans were re-examined to classify infarctions in the following patterns described by Bang et al: (1) single infarction; (2) multiple infarctions in a single arterial territory; (3) multiple small infarctions involving multiple arterial territories; and (4) multiple small and large disseminated infarctions.^[12]^ Disseminated intravascular coagulation (DIC) was defined according to the International Society on Thrombosis and Haemostasis standard by a score >5 following the criteria: platelet count (>100 G/L = 0, <100 G/L = 1, <50 G/L = 2); D-dimer (no increase = 0, moderate increase = 2, strong increase = 3); prolonged prothrombin time (<3 seconds = 0, >3 seconds = 1, >6 seconds = 2); and fibrinogen level (>1 g/dL = 0, <1 g/dL = 1).^[13]^ Leukemoid reaction was defined as a white blood cell count >50 G/L.^[14]^

All data were anonymized before collection. This study received the required legal approval from the appropriate French data protection committee (Commission Nationale de l’Informatique et des Libertés) (DE-2015–108).

### 2.2 Statistical analysis

All analyses were descriptive and carried out by using IBM SPSS Statistics software, version 20 (SPSS Inc., Chicago, IL). The study was observational and described clinical, radiological, and biological features of patients from our centers and in literature. Continuous variables are reported as median ± standard deviation and extreme values, and categorical factors as frequency and percentage. Statistical comparison was performed between characteristics of patients from our centers and from literature using the Mann–Whitney *U* test for continuous variables and Fischer exact test for categorical factors. A significant difference between both groups was defined as *P* < 0.05.

## 3 Results

During the study period, 17 patients with pancreatic cancer were admitted in the 4 centers after a cerebral event. Diagnosis of pancreatic cancer resulted from this hospitalization in 7 cases, and had already been known for a mean time of 5.4 months for the 10 others. Eighteen cases were found in the literature, all describing diagnosis of pancreatic cancer through management of the stroke (*P* = 0.001).^[10,15–22]^ Overall, 57% of patients were male, and median age was 63 ± 14 years, which ranged from 23 to 81 years. The patients in our series were older than in the literature, with median age of 70 versus 59 years (*P* = 0.02) (Table 1). In 93% of cases, pancreatic cancer was at a metastatic stage. All documented histologic types were adenocarcinoma except for 2: one patient had mucoepidermoid carcinoma and the other cystadenocarcinoma. Pancreatic location was fairly evenly distributed between the head (40%), and the body and tail (60%). All patients presented ischemic strokes, except for 2, who had transient ischemic attacks. No case of hemorrhagic stroke was found. Eighty-two per cent of our patients presented at least 1 vascular risk factor (apart from age) and 12% atrial fibrillation. Echocardiography performed in 4 cases revealed nonbacterial thrombotic endocarditis (NBTE) in 1 patient of our series, whereas it was found in 40% of documented cases of the literature. The fourth radiological pattern was the most common overall (54%) and 64% of patients presented disseminated cerebral infarctions (patterns 3 and 4). Silent other systemic infarctions were detected in 37% of the patients overall, with a predominance in patients from the literature (62 vs 25%; *P* = 0.01). Platelet counts were wide-ranging (180 ± 155 G/L), with a high median prothrombin time (19 ± 6 seconds, reference range: 12–16.5 seconds) and median D-dimer level (7600 ± 5 × 10^7^ μg/L) (Table 2). Median activated partial thromboblastin time and fibrinogen level were normal at 29 ± 8 seconds (reference range: 23–34 seconds) and 2.3 ± 1 g/L (reference range: 1.7–4 g/L), respectively. Two patients met the criteria of DIC. No leukemoid reaction was found: the median white blood cell count was 9 ± 6 G/L, ranging from 7 to 26 G/L. Median CRP and CA19.9 levels were elevated at 63 ± 43 mg/L and 47,000 ± 70,000 U/mL, respectively. Forty-three per cent of all the patients presented elevated liver transaminases. The 7 patients diagnosed with pancreatic cancer at stroke onset in our centers received chemotherapy as opposed to none in the literature (*P* = 0.02). Two of the 7 were treated with folfirinox (including 5-fluorouracil, oxaliplatin, and irinotecan) and 5 with gemcitabine. In our population, 10 patients were treated with anticoagulant therapy for secondary stroke prevention (3 with unfractionated heparin, 6 with low-molecular-weight heparin [LMWH], and 1 with a vitamin K antagonist). No cerebral ischemic recurrence was documented. All patients died after a median time of 28 ± 14 days after stroke onset, except for 2 with a mean follow-up of 4.5 months.

**Table 1:**
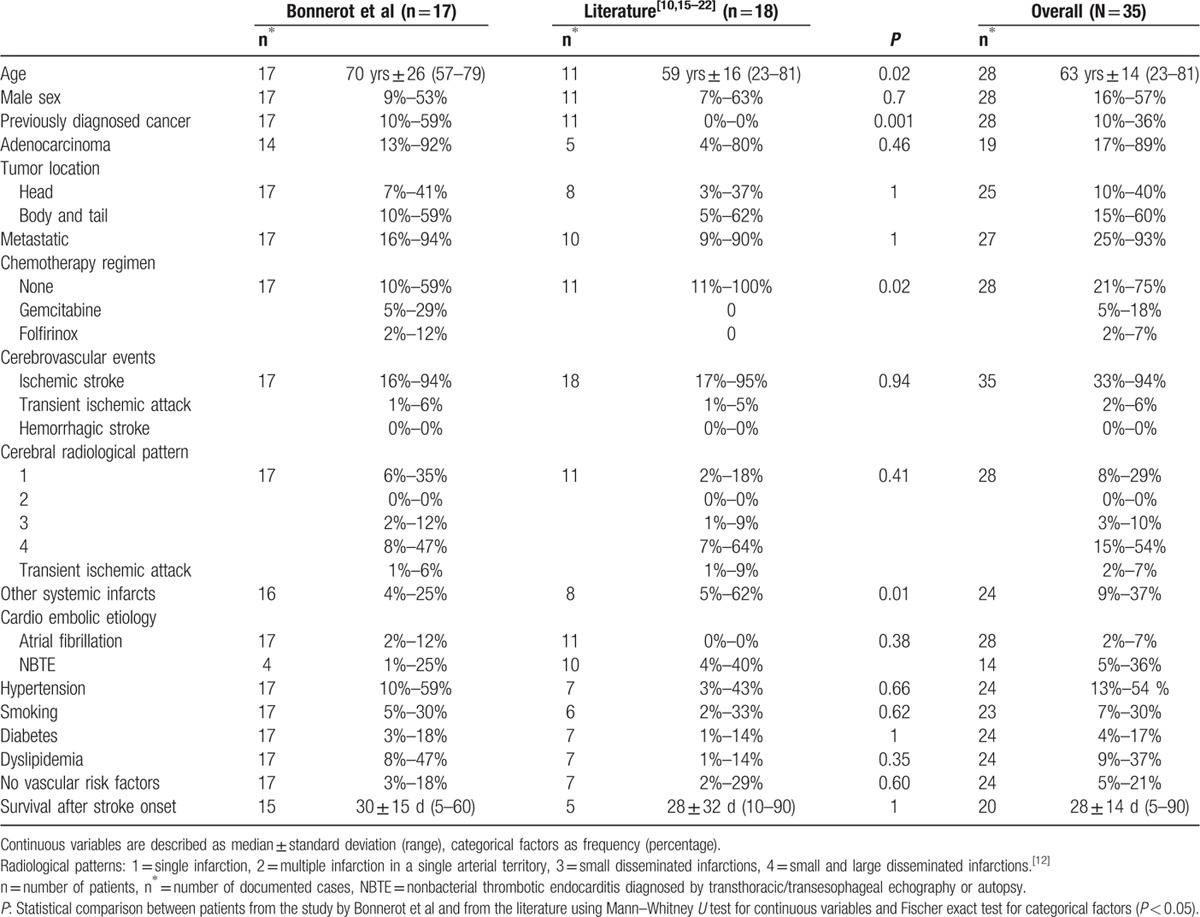
Demographic, clinical, radiological characteristics and outcome of patients with cerebral ischemic events and pancreatic cancer.

**Table 2:**
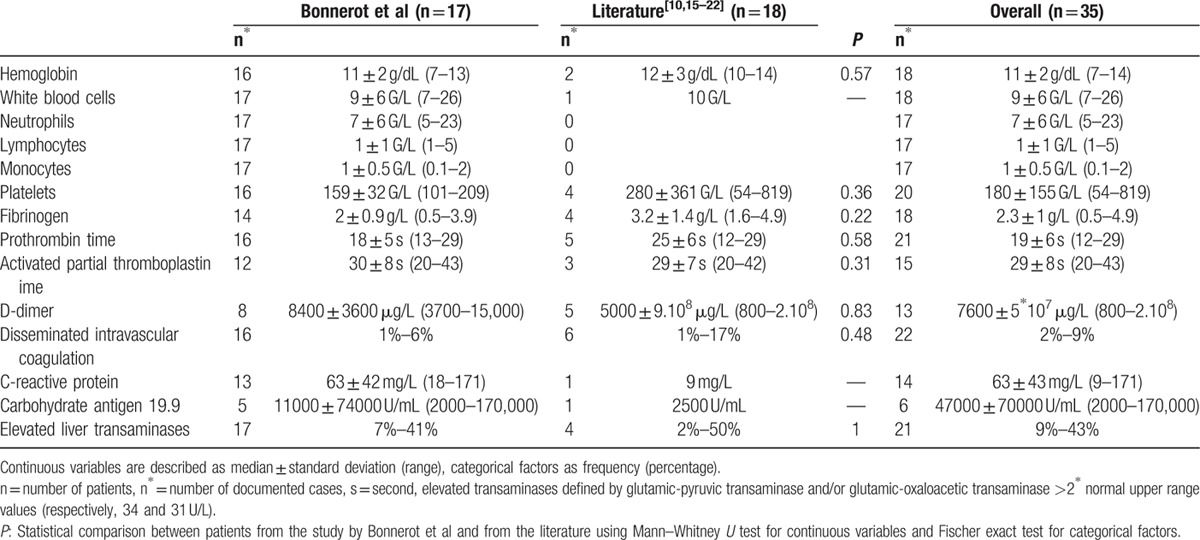
Biological characteristics of patients with cerebral ischemic events and pancreatic cancer.

## 4 Discussion

The incidence of arterial stroke complicating pancreatic cancer seems low. We identified only 17 cases in 4 centers specialized in the management of cerebrovascular diseases in a period of about 6 years. Whereas the retrospective design of this study and inclusion of patients only admitted in stroke units would certainly tend to underestimate the real incidence, an autopsy study found less than 1% of patients with pancreatic cancer presenting stroke.^[23]^ Prospective works have demonstrated that stroke is more frequent in patients with pancreatic cancer with a cumulative incidence of 3.4% at 3 months after diagnosis,^[9]^ and at higher risk during the first 6 months.^[8]^

All reported cases found in the literature describe stroke as “Trousseau syndrome,” which means that it precedes the diagnosis of the concealed pancreatic cancer. This diagnostic sequence has been the main focus of authors attempting to isolate special features of stroke to achieve earlier diagnosis and treatment of pancreatic cancer. Our series including consecutive cases shows that Trousseau syndrome led to cancer diagnosis in only one-third of patients. Stroke more often complicates an already known pancreatic cancer, in accordance to the results of prospective studies.^[9]^ Unfortunately, whatever the time of stroke onset, it almost always occurs at a metastatic stage.

**Figure 1:**
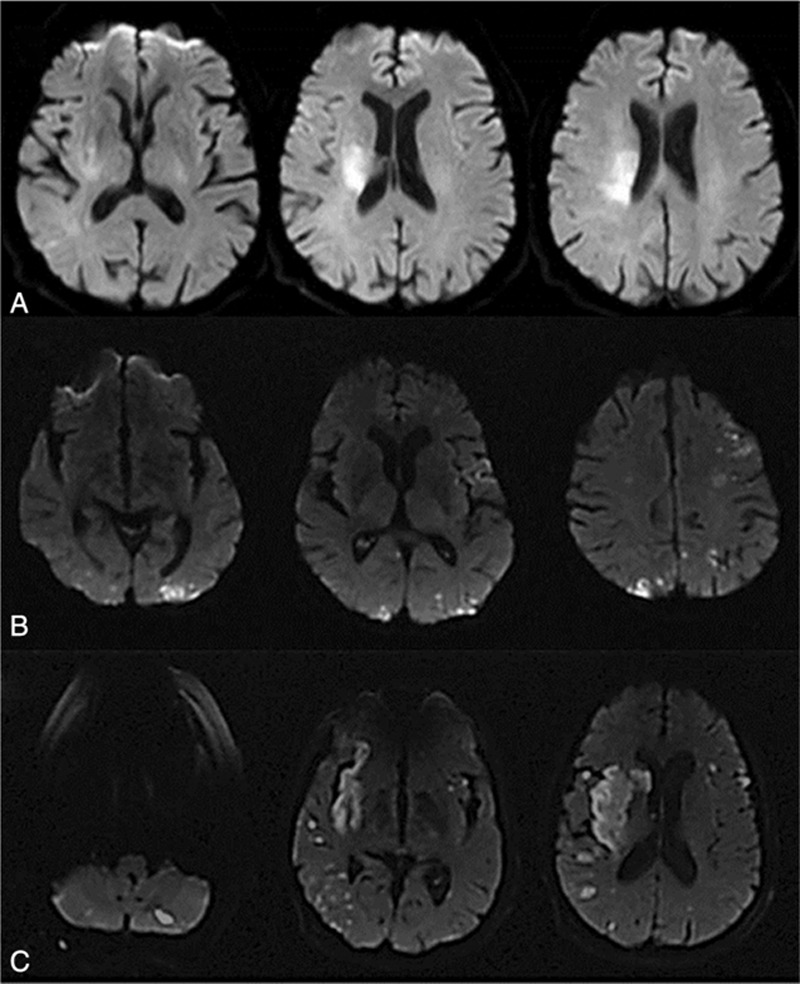
Brain diffusion-weighted magnetic resonance imaging showing radiological patterns of cerebral infarctions in patients with pancreatic cancer. A, Single infarctions (pattern 1); B, small disseminated infarctions (pattern 3); C, small and large disseminated infarctions (pattern 4). Patterns defined by Bang et al.^[12]^

All the patients included in this analysis presented ischemic events. Most presented infarctions in several arterial territories, many combining small and large lesions (Fig. 1).^[12]^ This particular radiological feature is common to adenocarcinomas and is highly suggestive of a thromboembolic mechanism.^[15,24]^ Many high-intensity transient signals were recorded on transcranial Doppler of patients with cancer-associated stroke, and these would appear to be correlated to D-dimer levels.^[25]^

**Figure 2:**
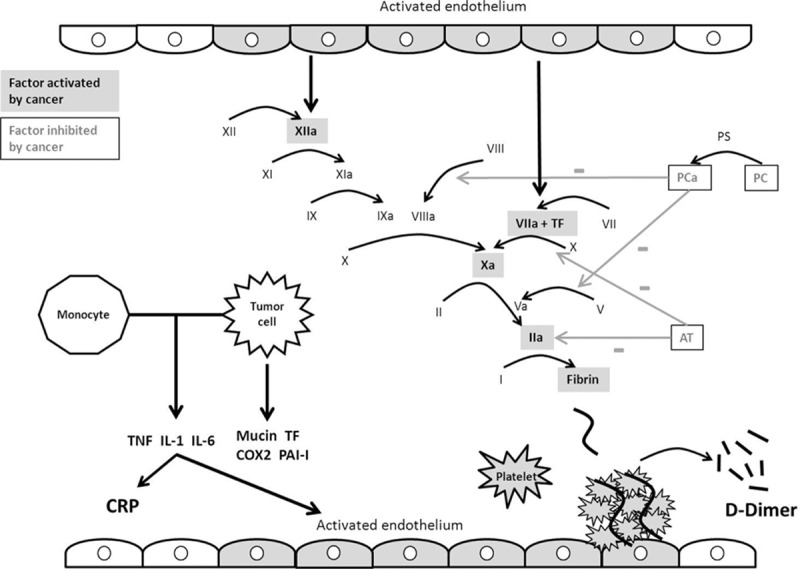
Intravascular coagulation disorders caused by cancer. a = activated, AT = antithrombin, COX2 = cyclooxygenase 2, CRP = C-reactive protein, IL = interleukin, PAI-I = plasminogen-activator inhibitor I, PC = protein C, PS = protein S, TF = tissue factor, TNF = tumor necrosis factor.

Several theories have been put forward to explain embolism in this setting.^[26]^ Our study found a high median prothrombin time, which would suggest a coagulation disorder. Literature cases also report a higher activated partial thromboplastin time in cancer-associated stroke patients.^[27]^ Activation of procoagulant factors (factor X, tissue factor, thrombin, and fibrin) and a decrease of anticoagulant factors (protein C and antithrombin) appear to be involved, with the latter more involved in venous thromboembolism (Fig. 2).^[28]^ A release of inflammatory cytokines (tumor necrosis factor, interleukin [IL]-1 and IL-6) due to interaction of monocytes with malignant tumor cells could result in damage to the arterial vessel walls, activation of platelet aggregation, and high levels of fibrinogen.^[1,19,29]^ The latter has been described as a biomarker of poor outcome.^[30]^ Cancer is also responsible for the production of mucin, which can activate coagulation and has been found within the cerebral infarction.^[31]^

Coagulation disturbance may be increased by activation of oncogenes (*RAS, MET, EGFR*) and inhibition of tumor-suppressor genes (*p53, PTEN*).^[32]^ Mutations of these genes lead to the production of procoagulant agents and especially tissue factor, with activation of the extrinsic coagulation pathway, and plasminogen-activator inhibitor I.^[32,33]^ Platelet aggregation is a result of the production of cyclooxygenase 2 and then thromboxane A_2_.^[34]^ The resulting hypercoagulable state not only causes thromboembolic events but also enables cancer growth and development. Thrombin and factor VII activate, respectively, the protease-activated receptors 1 and 2, promoting cancer cell proliferation, adhesion, motility, survival, and angiogenesis by induced synthesis of vascular endothelial growth factor. Fibrin production protects cancer cells from natural killer lymphocytes, facilitates migration through the vessel wall, and consequently supports tumor development.^[33,34]^

In addition, some studies have demonstrated that chemotherapy regimens are responsible for the occurrence of thrombotic events, including ischemic strokes.^[3,35]^ Li et al^[11]^ found an incidence of postchemotherapy ischemic strokes of 0.137% in 10,963 patients. Platinum-based chemotherapies, and especially cisplatin, are the most often incriminated, with an incidence of about 18% reported by Moore et al^[36]^ and Numico et al.^[37]^ The hypercoagulable state theory, with a decrease in protein C and increase in the von-Willebrand and tissue factor levels, is further supported. Other mechanisms have been put forward, including endothelium damage and renal dysfunction, causing hypomagnesemia and vasospasm.^[38]^ However, our patients were treated with oxaliplatin, which causes fewer thromboembolic events than cisplatin.^[36,39–41]^ Gemcitabine is more likely to result in systemic infarctions from thrombotic microangiopathy syndromes, associated with other signs like hemolytic anemia, thrombocytopenia, and renal dysfunction.^[42–44]^ Several cases of transitory thrombocytosis have been reported, but with rare thrombotic consequences.^[45]^ Ischemia induced by 5-fluorouracil is mainly described for the myocardium, but with the same mechanisms as for cisplatin (i.e., hypercoagulable state, vasospasm, and endothelium damage).^[46]^ Khorana et al developed a model to predict venous thromboembolism risk in patients treated with chemotherapy. Pancreatic location of the cancer is considered as a very high-risk criterion. Other risk factors are high platelet and leukocyte counts, low hemoglobin level, and elevated body mass index.^[47]^

We did not find any leukemoid reactions. Only 2 reported cases of pancreatic cancer patients describe this biological feature in the literature, but without thrombotic consequences.^[14,48]^ However, Khorana et al^[47]^ consider a leukocyte count of more than 11 G/L as a risk factor of venous thrombosis in the context of cancer and chemotherapy regimen. Interaction between leukocytes and platelets with stimulation on one other and forming leucocyte–platelet complexes has already been suggested to play a role.^[49]^ Platelet count could be high due to an inflammatory pathway or low due to coagulopathy. However, only 2 patients fulfilled the DIC criteria.^[19]^ Levels of D-dimer, a degradation product of fibrin and reflecting hypercoagulability, were found to be considerably elevated in our study. Radiological stroke patterns 3 or 4 with a D-dimer level over 1.11 μg/mL are thought to be associated with an oncological cause.^[12]^ CRP, a well-known biomarker of inflammation, was also consistently found to be elevated in our series. High liver transaminase levels were present in less than half of the cases, and often related to hepatic metastasis.

**Figure 3:**
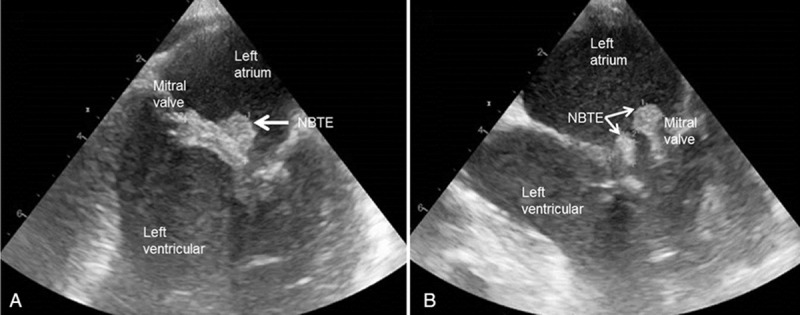
Transesophageal echocardiography, long-axis views, showing nonbacterial thrombotic endocarditis in a patient with pancreatic cancer. NBTE = nonbacterial thrombotic endocarditis.

Nonbacterial thrombotic endocarditis was diagnosed in one of our patients and in 4 cases in the literature.^[10,17,19,20]^ This common cause of embolism can be promoted by a prothrombotic state with deposition of fibrin and platelets as sterile vegetation on cardiac valves. However, transesophageal echocardiography was performed in only 2 of our patients (Fig. 3) (see Video, Supplemental Digital Content 1, which shows NBTE on mitral valve during transesophageal echocardiography). We suggest that patients should be systematically screened for NBTE in this context to confirm indication of anticoagulation therapy.^[50]^

**Figure 4:**
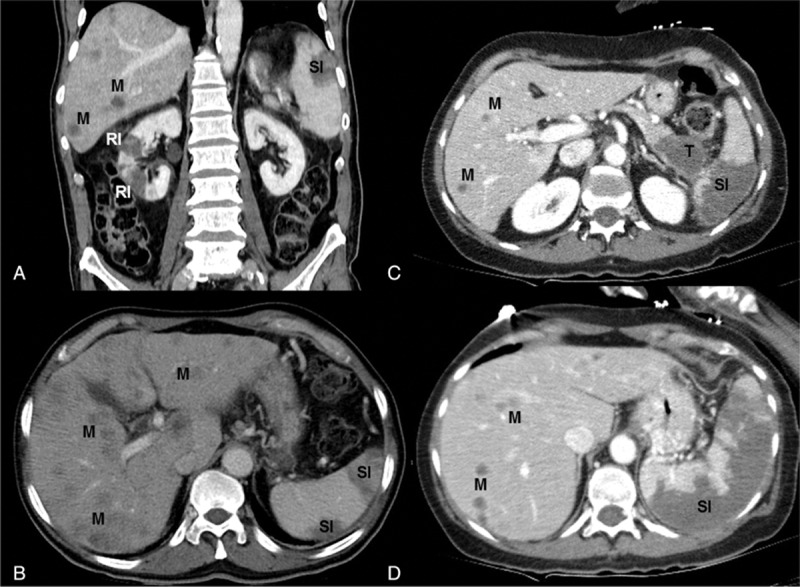
Abdominal CT scan showing pancreatic cancer (T), hepatic metastasis (M), renal (RI), and splenic infarctions (SI). A, B, CT scan without contrast. C, D, Contrast-enhanced CT scan. CT = computed tomography.

Other systemic infarctions were observed in only one-third of the patients, which raises the question of a specific cerebral mechanism of thrombosis (Fig. 4). Occurrence of cerebral vasculitis could promote cerebral infarction, but diagnosis requires more precise methods of angiography than 3-dimensional (3D) time-of-flight magnetic resonance imaging, which is usually performed.

On the other hand, median age of the patients we described was high, a single arterial infarction (radiological pattern 1) was found in 29% of cases, atrial fibrillation in 2 patients, and only 21% had no vascular risk factor. This raises the issue of whether the stroke was related to the cancer. Bang et al considers these as patients with “conventional stroke mechanisms,” with a pathogeny and distribution of etiologies indistinguishable from patients without cancer. They thus advocate preventive treatment “according to the stroke subtype” in these patients.^[12]^

Little is known about the best therapeutic strategy to prevent ischemic cerebral recurrences due to pancreatic cancer. Embolic mechanisms, coagulation disorders, and possible NBTE spontaneously appear as strong arguments to use anticoagulation therapy, but no reported long series of patients with pancreatic cancer supports this theory.^[50]^ Most of the studies about preventive treatment refer more to venous thrombosis recurrence than arterial infarction.^[51,52]^ The use of LMWH emerges as the most effective and safest therapeutic attitude to decrease stroke recurrence.^[53]^ Anticoagulation prevents thromboembolic events related to cancer and chemotherapy regimens, but unfortunately no gain on cancer development has been demonstrated.^[54]^ We confirm poor outcome with rapid decline in nearly all patients after stroke onset due to progression of cancer.^[55]^

Finally, the most relevant criteria that emerge to characterize stroke due to pancreatic cancer are as follows: a radiological pattern 4, a high D-dimer and CRP level, and an elevated prothrombin time. These investigations are usually performed in stroke patients, whatever the suspected etiology, and are thus easy to check. However, these features are not specific to the pancreatic location, as they are described in stroke patients with various oncologic diseases.^[56]^

The main limitation of this study is the retrospective design. Even if collecting data from a series of consecutive patients is more valid than isolated reported cases, we lack systematic assessment of the patients during the stroke management. For instance, we believe that the actual rate of NBTE and number of patients with cerebral vasculitis due to inflammatory process was higher. In addition, cerebral ischemic recurrences could be greatly underestimated due to the difficulty of conducting follow-up in this context. Finally, faced with the scarcity of the condition, multicenter studies are required with carefully standardized clinical, radiological, and biological assessment to better understand the pathogeny of cerebral infarctions related to pancreatic cancer, and to identify patients at high risk of this complication. Screening for specific biomarkers could be an interesting approach.

In conclusion, cerebral ischemic events are a complication occurring more often at an advanced stage of pancreatic cancer already diagnosed at stroke onset. A radiological pattern with disseminated infarctions of different size, a high D-dimer and CRP level, and an elevated prothrombin time are strong indicators of cancer-related stroke, but not specific to the pancreatic location. In this context, the patient should be systematically explored for NBTE. Anticoagulation therapy, especially LMWH, and etiological treatments remain the best strategy to prevent cerebral ischemic recurrences. Further studies are needed to determine which of these vulnerable patients are at risk to present ischemic stroke, and the best therapy to prevent this dramatic complication.

## Acknowledgments

The authors thank Sophie Marchal (Hospital of Verdun/Saint-Mihiel, Stroke unit, France), Raphael Demettre (Hospital of Bar-le-Duc, Stroke unit, France), and Alexandrine Larue (Hospital of Epinal, Stroke Unit, France) for their help to collect data.

They also extend their thanks to Felicity Neilson, Matrix Consultants, for having reviewed the English language with scientific expertise.

## Supplementary Material

Supplemental Digital Content
